# The evolutionary replacement of restriction-modification by Ssp antiviral systems is associated with the distribution of prophages in the major clonal group of *Acinetobacter baumannii*

**DOI:** 10.1128/mbio.02135-25

**Published:** 2025-08-18

**Authors:** Antonio Moreno-Rodríguez, Alejandro Rubio, Andrés Garzón, Younes Smani, Antonio J. Pérez-Pulido

**Affiliations:** 1 Andalusian Centre for Developmental Biology (CABD, UPO-CSIC-JA), Seville, Spain; 2 Department of Molecular Biology and Biochemical Engineering, University Pablo de Olavide 200143, Seville, Spain; University of Pittsburgh, Pittsburgh, Pennsylvania, USA

**Keywords:** anti-phage system, defense system, CRISPR-Cas, restriction-modification, Ssp, *Acinetobacter baumannii*

## Abstract

Bacteriophages kill almost half of the world’s bacterial cells every day. In response, bacteria have developed anti-phage defense mechanisms that number in the dozens. Individual defense systems are gained and lost by genomes of the same species, depending on their fitness advantage. Thus, the combination of defense systems presented by the individual genomes may differ, and the collection of all of them is what is known as the pan-immune system of the species. Here, we have analyzed thousands of genomes of the bacterium *Acinetobacter baumannii*, an opportunistic pathogen of humans of great clinical concern, and we have found 81 different defense systems. By analyzing how these systems combine, we have found that more than half of the genomes lack the universal DNA-methylating restriction-modification systems and harbor an alternative innate Ssp system that performs DNA phosphorothioate modification. The presence of one or the other innate system may modulate the evolution of the genomes of this species, causing them to present a different profile of phages integrated into the bacterial genome. In fact, the most widespread strain of this bacterium worldwide, global clone 2, showed an Ssp system as its distinctive feature. We have also observed that the presence of many other defense systems is associated with the presence of a higher number of prophages, which could be because the prophage carries the system, or because the bacterium would not need these systems in environments where the phage is absent.

**IMPORTANCE:**

*Acinetobacter baumannii* is a bacterium of great concern in clinical contexts due to the plasticity of its genome and its resistance to antibiotics. Its cells are infected by a multitude of bacteriophages, and the bacterium defends itself with dozens of different defense systems. Here, we have analyzed the complete defensome of thousands of genomes of the species and found that more than half of the genomes do not have universal restriction-modification systems, which are replaced by another innate labeling and restriction system. Furthermore, these genomes belong to the international clone of the bacterium that causes the most concern in hospitals.

## INTRODUCTION

Bacteriophages, also known as phages, are viruses that infect prokaryotes, which outnumber bacteria by a ratio of 10 to 1 (1). Phages usually attach to bacteria through surface structures such as pili or flagella and, using membrane proteins as specific receptors, inject their genome into the bacterial cell (2). As a result, they are often highly specialized, and a specific phage usually recognizes only bacteria of a certain species or even only strains that have a particular surface protein involved in the attachment phase or entry of the genome into the cell.

Bacteria defend themselves against phages using different systems. First, they present mechanisms that block the adsorption of the phage into the bacterium, thus preventing the entry of its genome (3). Once the phage genome enters the bacterium, there are three main defense systems, which have been known for decades. By far, the most studied are the innate restriction-methylation (R-M) systems that appear in 74% of bacterial genomes (4), with no taxonomic group of bacteria or archaea lacking them entirely (5). They are based on prior labeling of the bacterial genome with methyl groups to subsequently destroy the unlabeled genome of possible viruses entering the bacterium by means of site-specific endonucleases (6). On the other hand, CRISPR-Cas systems are adaptive immunity systems, discovered in the 2000s (7, 8) and appearing in 39% of bacterial genomes and 90% of archaea (9). These act similarly to a vaccine, as they can store fragments of phage genomes from previous infections called spacers, which allow them to act more quickly and efficiently against future infections of the same phage. Spacer formation appears to be enhanced in cells that also carry R-M systems, as these limit phage replication through phage DNA degradation, and cleavage products can be captured by the Cas adaptation machinery (10, 11).

Finally, abortive infection (abi) systems have been initially described as a programmed cell death induction mechanism triggered by phage infection, preventing the spread of the phage within the bacterial population (12). These systems are currently considered more of a late response to infection, in which bacteria trigger reversible growth arrest that would affect the replication of new phages. Thus, this bacteria-phage battle can lead to the death of the cell, depending on the context (13, 14). An example of this is the CBASS system (cyclic oligonucleotide-based antiphage signaling system), which responds to viral infection based on a signaling process (15). Another type of abi-associated systems is toxin-antitoxin, which adds to the arsenal of bacterial defense mechanisms in genomes carrying the two components of these types of systems (16).

All these antiviral systems are unevenly spread in bacteria, and even within the same species, they may appear in some genomes and not in others (17). Thus, they are part of the accessory genome of the species and are often dispersed by horizontal transfer (18). In fact, many more anti-phage defense systems have been discovered in recent years. As a result, we can define the pan-immune system of a species, or defensome, as the set of defense systems present in all the genomes of this species (19, 20).

R-M is a type of innate system in bacteria that serves as a defense against virtually any phage lacking the system’s methylation pattern. Uncommonly, there are bacteria that lack them and instead have other innate systems based on a different DNA labeling pattern, such as Ssp, BREX, and Dnd (21, 22). SspABCD-SspE and SspABCD-SspFGH systems, variants of the Ssp system with different effector genes, are among the most studied, and they confer high protection in *Escherichia coli* against coliphages (23–25). They label the resident DNA by modifying the sugar-phosphate backbone of it, replacing the non-bridging oxygen with sulfur in a phosphorothioation reaction.

All these systems are usually found in genomic islands and archipelagos, integrated in hotspots, which allows for easier discovery (26–28). Based on this particularity, 29 groups of antiviral genes were recently discovered, involved in new defense systems with functions of RNA editing, satellite DNA retron synthesis, ATPases, and DNases, which are generally classified as Gao systems (29, 30). Phages themselves are sometimes involved in the mobilization of these defense systems and may even carry hotspots with several of them, which can make them interact positively and negatively with respect to other competing phages (18, 31).

Phages, in turn, defend against the bacterial defense systems with anti-defense systems in an ongoing arms race (32, 33). In addition, phages can co-opt bacterial antiviral systems, promoting their dissemination while using them to defend against other phages and increase their competitiveness (34).

Given that the different systems of the defensome of a bacterial species are found in its accessory genome, a representative number of genomes is required to perform the study at the pangenome level. Bacteria usually maintain their accessory genes only if they provide an advantage in the environment in which they are found; otherwise, they would act as useless ballast, especially in large populations that allow for rapid counterselection (35). Thus, if bacterial cells are subject to selection pressure by a phage from a specific environment, they will tend to maintain specialized defense systems against it. Moreover, if bacteria in turn have accessory genes that facilitate phage infection, such as membrane proteins that act as adhesion sites or entry receptors, the maintenance of these defense systems becomes even more important (36).

Here, we have studied the defensome of the opportunistic gram-negative pathogenic bacterium *Acinetobacter baumannii*, based on a previous pangenome of almost 9,000 genomes. This bacterium has been classified as a critical priority by the World Health Organization due to its high rate of antimicrobial resistance and the urgent need to develop new drugs to combat it. We have found 81 different phage defense systems, of which the most prevalent are the Ssp and Gao_Qat systems (including a protein with a domain similar to 7-cyano-7-deazaguanine synthase associated with an ATPase and a DNase), which are present in the genomes most frequently isolated in hospital infections, global clone 2 (37). The remaining clonal groups substitute the Ssp with R-M systems and have a lower number of integrated prophages in their genomes. Furthermore, we have performed a machine learning analysis and predicted species-specific prophages that are avoided by certain defense systems, as well as prophages that co-occur with specific defense systems. These findings suggest that these defense systems may be used in battles between phages, where bacteria would be the battlefield.

## RESULTS

### *A. baumannii* genomes have 81 different defense systems

To study the defense systems against phages infecting *A. baumannii*, 8,929 bacterial genomes isolated worldwide were used (Table S1). These genomes constitute a pangenome of 39,947 genes, with 2,220 core genes and 37,727 accessory genes.

**Fig 1:**
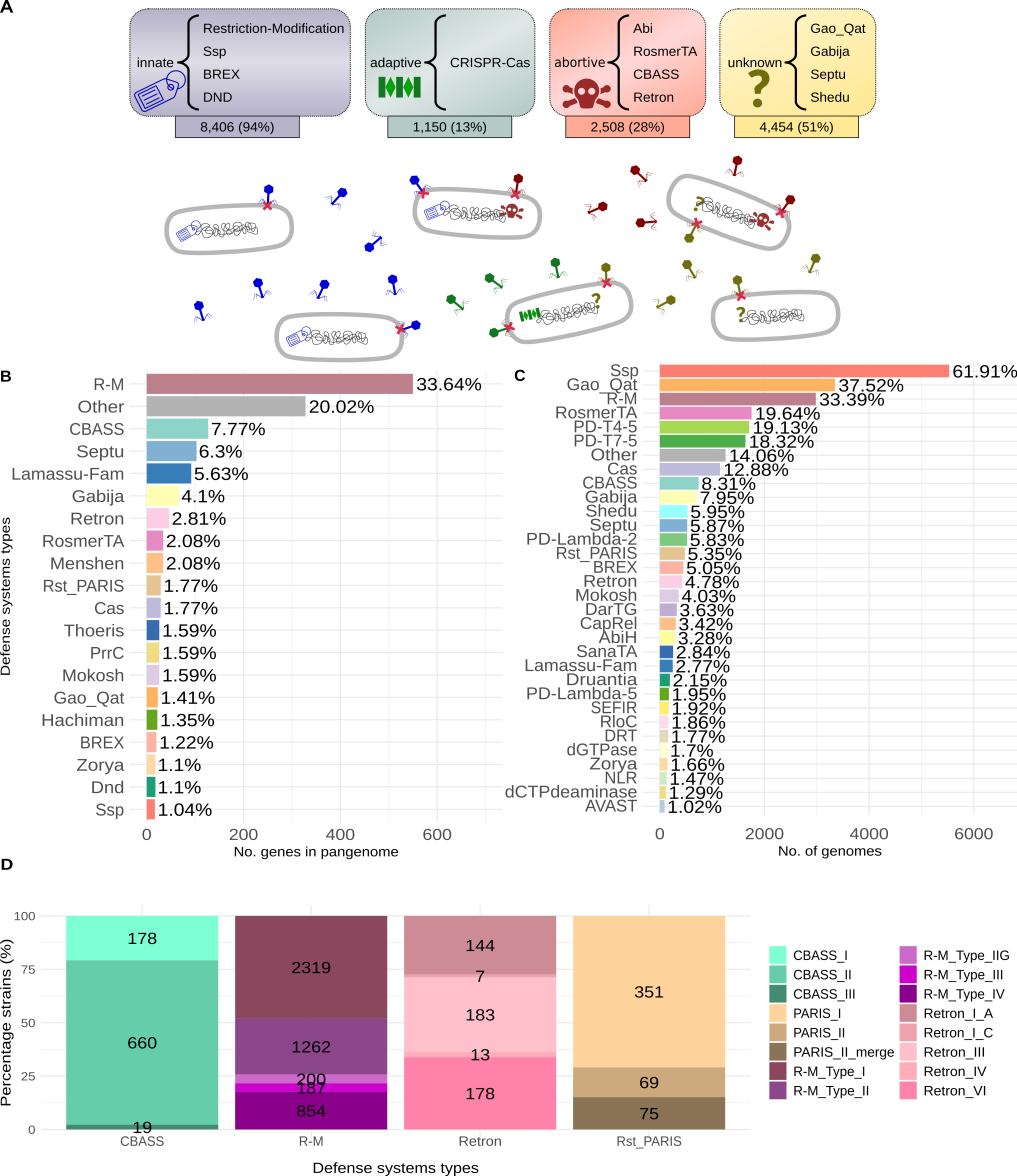
*A. baumannii* defensome. (**A**) Most frequently encountered defense systems grouped by type: innate, adaptive, abortive, and unknown. Under each group is the number of genomes that have any system of this type, as well as the relative frequency of genomes. These results show that not all genomes have all systems, which would suggest that each one could be specialized in different phages. In this way, in environments where there are phages that are sensitive to a certain defense system, this defense system should be prevalent in the bacteria, while the rest would be dispensable. Consequently, the bacteria would improve their fitness without the need to maintain the genes of defense systems that are not necessary in that environment. (**B**) Absolute and relative frequency of genes belonging to each system with respect to the total number of defense genes in the pangenome. (**C**) Absolute and relative frequency of genomes that possess each one of the systems, ordered from most to least frequent. (**D**) Number of genomes having each defense system subtype, for some of the systems.

Based on this pangenome, 81 different complete defense systems were found, with all but 98 genomes analyzed encoding at least one of them. Genomes in which complete defense systems were not found belonged to different clonal groups and showed greater fragmentation than average (number of sequences = 267.38 ± 263.05 and NG50 = 149,225 ± 563,534; see Materials and Methods). However, these genomes did contain isolated defense genes. Given that the absence of defense systems was not clear in these genomes, we decided to exclude them from further analysis. Thus, the complete systems found can be classified into four groups according to their mechanism of action: innate, adaptive, abortive, and unknown systems (Fig. 1A). The systems most prevalent in the defensome of *A. baumannii* were innate (present in 94% of genomes), followed by systems with unknown mechanisms (present in 51% of genomes).

A total of 1,619 different genes were identified as part of the diverse defense systems (4% of the pangenome). These genes, which were mainly accessory genes, were not present in all the genomes and collectively form the defensome of the species (Table S2). Only 11 core genes (*prmB*, *fokIM*, *cysH*, *yjjV*, *fstK*, *pkn5*, *htpG*, *mdcH__2*, *air*, *eptA__3*, *mgtA__3*), present in virtually all genomes, were predicted to assist certain very rare configurations of the defense systems BREX, CapRel, Cas, Mokosh, PD-Lambda-5, R-M, Ssp, and Gao_Qat.

The R-M systems presented the largest collection of different genes, constituting 33.64% of the defensome (550 different genes). However, only 33.39% of the genomes analyzed had a full version of either of the subtypes of these innate systems (Fig. 1B and C). It should be noted that R-M systems, as well as the CBASS and Retron toxin-antitoxin systems, were subdivided into different subtypes, which may mean that they have slightly different mechanisms of action. For example, there are 2,319 genomes with R-M systems type I and only 187 with type III, or 854 with type IV (Fig. 1D).

None of the identified defense systems were present in all *A. baumannii* genomes, which highlights their diversity across different bacterial strains. On average, each individual genome had 3 ± 1.90 different defense systems. The other innate system, Ssp, was composed of a total of 17 genes and appeared in 61.91% of the genomes analyzed, making it the most frequent of all defense systems. In most cases (5,518 out of 5,530), the nuclease of this system, *sspE*, was not found alongside the other genes. However, the system was located in a genomic region with other genes that encode restriction endonucleases (Fig. S1). Ten of the genes in this region had the remarkable feature of appearing in virtually all strains of global clone 2 of *A. baumannii* and did not appear in the rest of the clones.

Other lesser-known defense systems, such as Gao_Qat, appeared in 37.52% of the genomes, suggesting its possible relevance.

**Fig 2:**
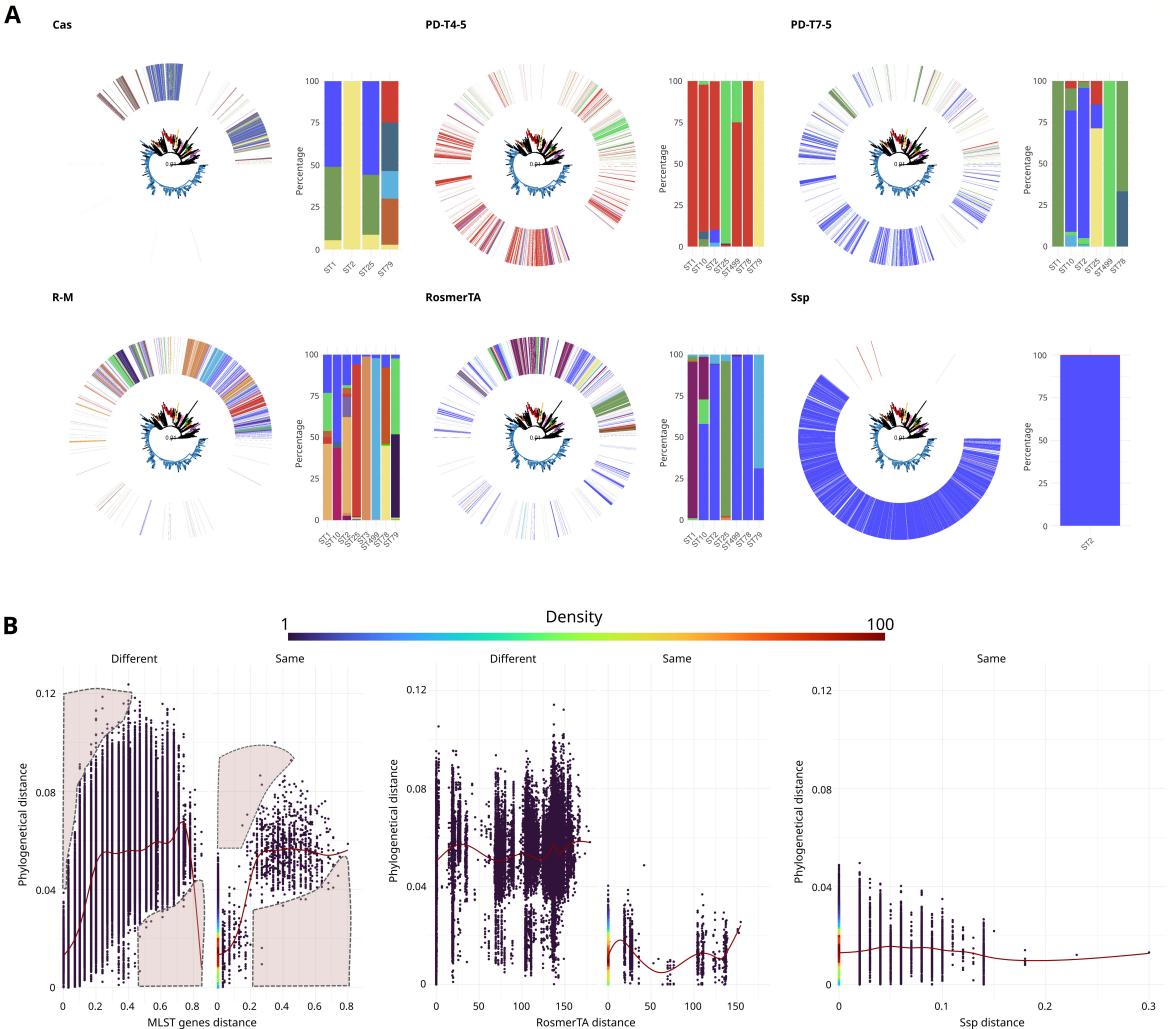
Phylogenetic distance versus sequence divergence of the most common defense systems. (**A**) Distribution of sequence variants (95% identity threshold) of genes from six of the most common defense systems throughout the phylogeny of the species, together with the proportion of each variant in the most common ST groups. The phylogeny used is the same as in Fig. 4D. (**B**) Dispersion of phylogenetic distance versus sequence divergence of genes belonging to different defense systems. Each point corresponds to the comparison of two genomes, and the color (density) highlights the number of points in a given region. Different = the two genomes belong to different ST groups; Same = the two genomes belong to the same ST. The results are shown from left to right: for core genes used in MLST classification (multilocus sequence typing; Pasteur scheme) and subjected to vertical transfer (control), RosmerTA system, and Ssp system. In the control, four areas have been marked with shapes delimited by a dashed line, which highlight “avoided areas” when analyzing genes subjected to vertical transfer.

It is known that defense systems can be transferred and acquired through horizontal gene transfer. To verify that this process also occurred in the most common defense systems of *A. baumannii*, the distribution of their variants along the phylogenetic tree of the species was examined. It was found that the same sequence variant of a system could be found in evolutionarily distant genomes (Fig. 2A). To assess the extent of this dispersion of sequence variants in defense systems, we compared their nucleotide sequences with the phylogenetic distance of the genomes in which they were found. Using conserved genes from the species as a control, we found that their sequence divergence correlated positively with phylogenetic distance, thus confirming vertical transfer of these genes (Fig. 2B). This comparison revealed avoided areas, meaning that the same gene variant did not appear in distant genomes, and highly divergent sequences did not appear in evolutionarily close genomes. In contrast, genes associated with defense systems showed a different behavior, with no positive correlation. Now, the same sequence variant can appear in evolutionarily distant genomes, and genes with very different sequences appear in closely related genomes. This result supports horizontal gene transfer of defense systems in *A. baumannii*.

### Different innate defense systems do not usually appear together in the same genome

Since the different defense systems of *A. baumannii* are part of its accessory genome, we wanted to know if these were randomly distributed or if there was any coincidence or incompatibility of systems in the same genome.

**Fig 3:**
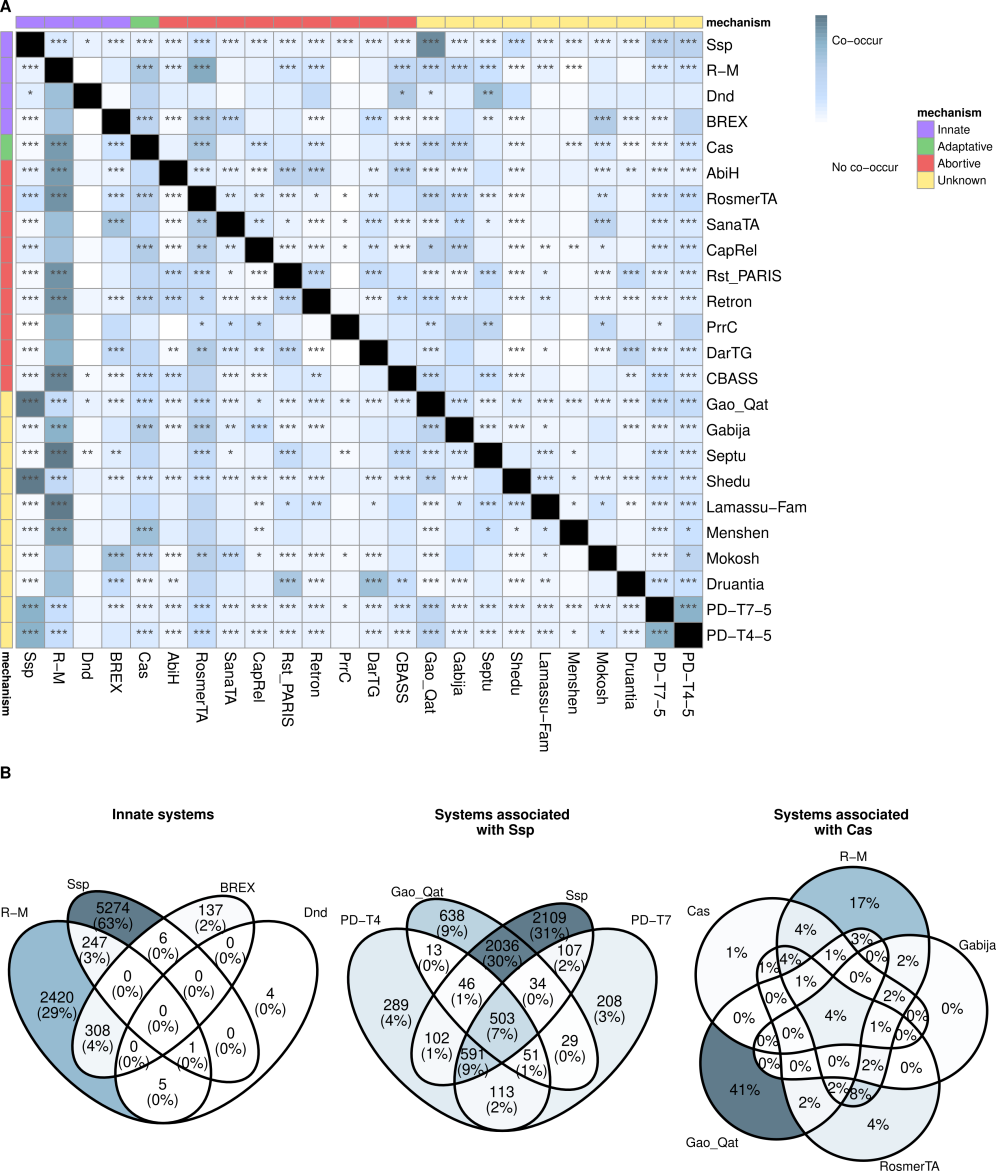
Coappearance of defense systems. (**A**) Coappearance of defense systems that appear in at least 1% of strains from the pangenome (97 genomes). The diagonal above shows the proportion of genomes that, having the row system, also have the column system, and the diagonal below shows the proportion of genomes that, having the column system, also have the row system. Fisher’s test was used to determine whether there is a significant association (positive or negative) between two systems (*P*-value <0.001, “***”; *P*-value <0.01, “**”; *P*-value <0.05, “*”). (**B**) Multiple co-occurrences of sets of defense systems: innate systems, systems frequently associated with Ssp, and systems frequently associated with CRISPR-Cas. In the first two cases, the number of genomes is shown together with the relative frequency, and in the last case, only the relative frequency is shown for simplicity.

Analysis of the systems that co-occurred in the genomes revealed a predominant association of many of the most frequent defense systems with R-M (Fig. 3A). The CRISPR-Cas and RosmerTA systems are particularly noteworthy. Thus, the presence of one of these systems is usually also accompanied by the presence of an innate R-M system. On the contrary, when systems such as Ssp (the other innate-tagging system), Gao_Qat, Shedu, and PD-T systems (PD-T4-5 and PD-T7-5, which appear together 3 out of 4 times) appear in the genomes, R-M systems are rarely found.

Notably, the four innate systems of *A. baumannii* appear to be mutually exclusive, especially the R-M and Ssp systems (Fig. 3B), with the latter appearing strongly associated with the aforementioned systems not coappearing with the R-M systems: Gao-Qat and PD-T systems. However, the R-M systems present a high coappearance with the CRISPR-Cas systems, and 84% of genomes that have CRISPR-Cas systems also have R-M systems. In addition, genomes having CRISPR-Cas systems also tend to have RosmerTA (56%), Gabija (37%), and Gao-Qat (34%) systems with a high frequency (Fig. 3B). Since the R-M systems are known to synergize with CRISPR-Cas (10, 11), some of the other three mentioned systems could also help them in some of their steps.

### R-M systems appear to be more efficient than Ssp in avoiding phages

The defense systems found allow the bacteria to defend themselves against phage infection. To test their expected efficiency and specificity, we searched for phages integrated into the genomes of *A. baumannii*. Of the more than 2,000 different prophages found in the pangenome, only those that were present in at least 0.1% of the total number of genomes (group “total prophages”) and that appeared most frequently in the major clonal groups of the species (group “frequent prophages”) were taken for further analysis. These sets of prophages were searched in all bacterial genomes.

**Fig 4:**
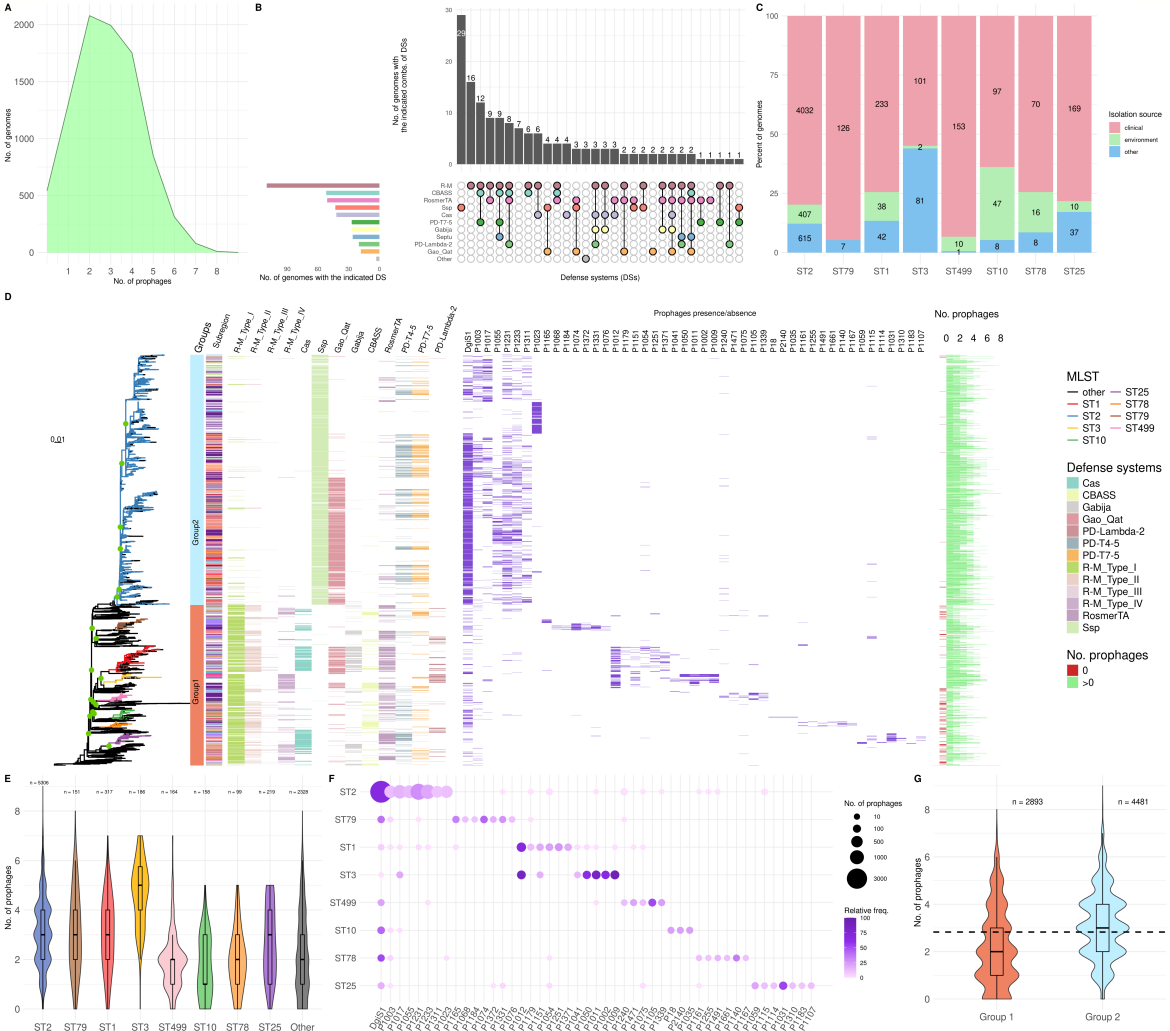
Prophages in the genomes of *A. baumannii*. (**A**) Number of genomes that have a given number of total prophages. (**B**) Number of prophage-lacking genomes differentiated by the defense systems they carry and their combinations. (**C**) Number of genomes of each of the most frequent clonal groups of the species, differentiated by their isolation site. (**D**) Molecular phylogeny of all the genomes analyzed, highlighting the most frequent clonal MLST groups (multilocus sequence typing; Pasteur scheme). Nodes separating the main MLST groups and others with a bootstrap value higher than 80 are shown with green dots. Next to the tree, from left to the right, it shows in order distribution of the two main groups in the phylogeny (group 1 and group 2 genomes), geographical location of the isolate separated by subcontinent, presence-absence of the most frequent defense systems, presence-absence of the most frequent prophages by clonal group, and number of total prophages (0 prophages is shown in red). Details on the distribution by subcontinent of the different MLST groups can be found in Fig. S2. (**E**) Distribution of the number of total prophages in the different clonal groups. Significant differences between the numbers of phages in each clonal group were determined using the Kruskal-Wallis test (*P*-value = 4.01e − 214) in order to test the different prophage distribution belonging to each clonal group. (**F**) Presence-absence (abundance) of the most frequent prophages in the different clonal groups. (**G**) Distribution of the number of total prophages in the two main groups of genomes (group 1 and group 2) shown in the phylogeny with the average number of total prophages. Significant differences between the numbers of phages in each clonal group were determined using the Wilcoxon test (Wilcoxon, *P*-value = 2.63e − 141).

The first thing to note is that there was an average of 2.83 ± 1.55 prophages per genome (Fig. 4A). However, 183 genomes did not have any prophage, and 1,613 did not have any of the 47 frequent phages. Most of these 183 genomes had R-M systems (60%), demonstrating the efficiency of these systems (Fig. 4B). Of the remaining genomes lacking prophages, 43 had the alternative innate Ssp system (with only three of them also having R-M systems), and nine had innate BREX systems (with five genomes also having R-M systems). This suggests that the R-M and Ssp systems may be alternative innate systems, while the BREX systems could be synergistic with the R-M systems. Remarkably, seven of the genomes without prophages do not exhibit any defense system. This suggests that they either have as yet undescribed defense systems or belong to bacterial strains that have not been subjected to selection pressure from phages.

The defense systems of a bacterial species are part of what is known as the pan-immune system. In support of this concept, the same system should be scattered throughout the pangenome of the species, and specific defense systems could be acquired or lost by genomes of the species, with the global population being a reservoir for all of them. However, certain defense systems could be linked to clonal groups of the bacterium, if these offer them some advantage in their environment, such as defense against the phages they may encounter there. To analyze this, we studied the distribution of the most frequent defense systems according to the phylogenetic relationships of the genomes containing them, with special emphasis on the most frequently isolated clonal groups of *A. baumannii*, which have been mainly isolated in clinical environments (Fig. 4C and D). In addition, we analyzed the presence/absence and abundance of frequent prophages according to their phylogenetic relationships and their association with frequent defense systems, to test the effectiveness of defense systems. It should be noted that the genomes analyzed had been isolated from various locations around the world, revealing low bias due to geographical origin (Fig. 4D; Fig. S2).

The prevalent ST2 clonal group and other phylogenetically close groups (group 2) seem to have almost exclusively the Ssp system in their genomes (96%), while the rest of the groups, evolutionarily distant from ST2, present R-M systems instead (group 1). Group 2 also presents, with less frequency, the other defense systems seen as associated with Ssp, the most common being the Gao-Qat and the PD-T systems (45% and 26%, respectively). Meanwhile, the rest of the genomes present a greater heterogeneity of defense systems, although always lacking Ssp systems. This correlates with the fact that there are more prophages on average in group 2 than in other less prevalent clonal groups (Fig. 4E). One of the most frequent prophages in this clonal group is DgiS1 (Fig. 4F). If we consider that the defense island in which the prophage is integrated is also the site of integration of the genes of the Gao_Qat system, this could point to the fact that these defense systems could be associated with specific prophages of the bacterium, which would use them as weapons to fight against other different prophages. In fact, genomes that present an Ssp or Gao_Qat system as the only defense system presented three to four integrated prophages (more than the average of all genomes). Another prophage in the ST2 group is P1023, which appears in genomes with Ssp, but not with Gao-Qat or PD-T.

On the other hand, although many of the different prophages of the species appear in different clonal groups, there are prophages that have a greater frequency in certain taxonomic groups, and even almost exclusively in some of them. This shows how clonal groups with specific combinations of defense systems tend to have different prophage profiles. Thus, prophage P1012 only appears in the ST1 and ST3 groups, prophage P1031 in the ST25 group, prophages P1002, P1009, P1011 and P1050 are more prevalent in the ST3 group, and prophage P1105 only appears in the ST499 group (Fig. 4F).

Genomes in group 1 do not appear to have a high proportion of CRISPR-Cas and CBASS systems (1,045 and 612 genomes, respectively), co-occurring only in 148 genomes. Of those with CRISPR-Cas, 83% also had R-M systems, and of those with CBASS, 86%. However, the alternative innate Ssp system did not coappear with CRISPR-Cas systems in any of these genomes, and only in five genomes did it coappear with CBASS systems across the entire pangenome.

These results suggest a highly differentiated defense system profile between both clonal groups of *A. baumannii*, with the Ssp system being specific to group 2 and the R-M systems to group 1.

These two groups show distinct prophage profiles and also show a quantitative difference in the number of prophages, with an average of 2.18 ± 1.63 prophages in group 1 and 3.12 ± 1.43 prophages in group 2 (Fig. 4G). This could be due to the homogeneous profile of the defense systems or to the low number of systems in group 2. Finally, we wanted to know whether other accessory genes, such as those involved in antibiotic resistance, appeared differentially in both groups. We found that the genomes of group 2 had a high number of genes of this type (15.21 ± 6.29). However, in group 1, there were fewer (10.55 ± 6.62), and even in half of their genomes, the number of antibiotic resistance genes was around 5 (Fig. S3).

### The presence of certain defense systems is associated with specific prophages

**Fig 5:**
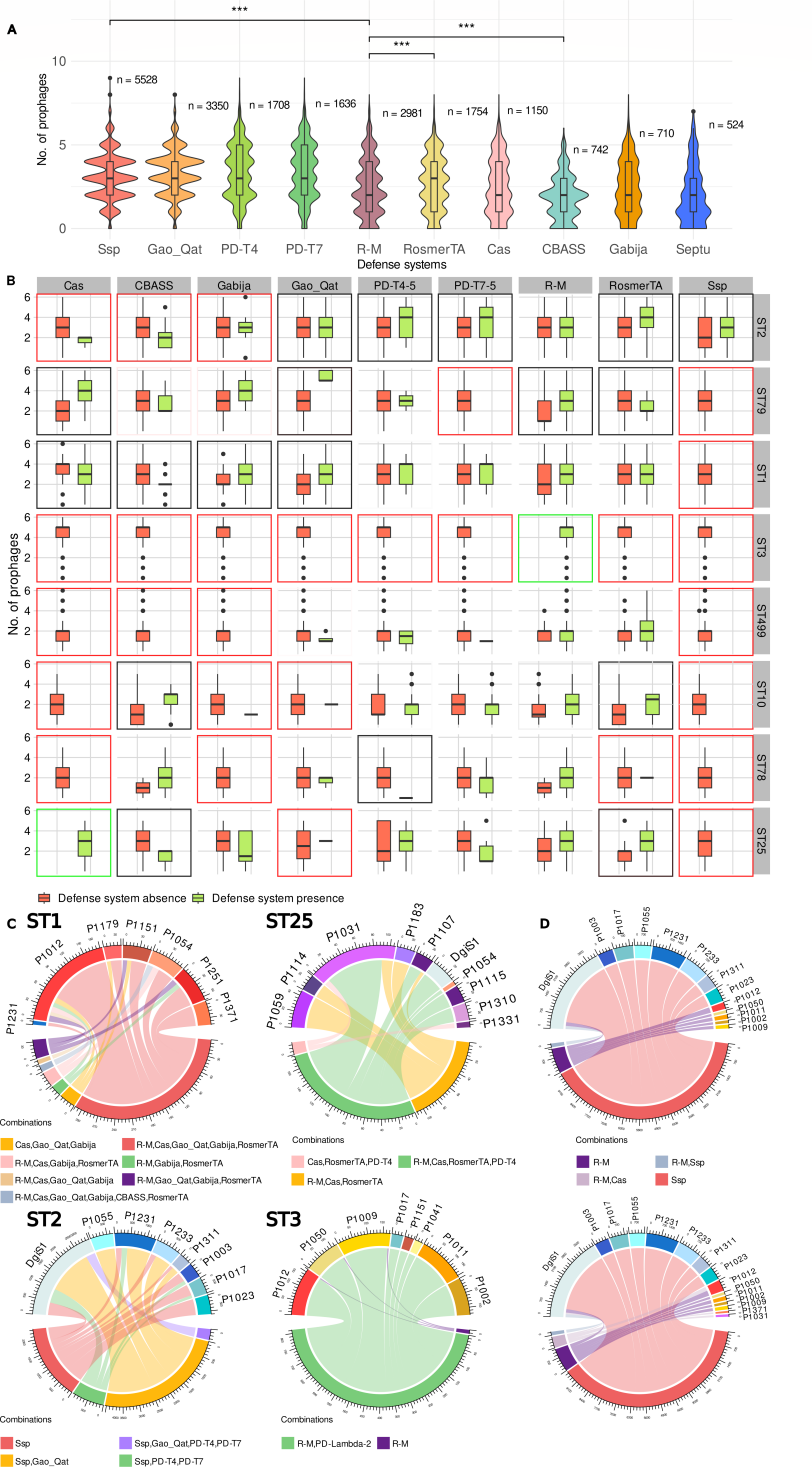
Number of prophages according to the defense system possessed by the bacterium. (**A**) Distributions of the number of prophages according to the defense system of the bacterium. Only the most relevant comparisons are shown, such as the main defenses of each group, Ssp and R-M, or the systems associated with a different number of prophages within each group (paired Wilcoxon test between groups; *P*-value <0,001, ***). (**B**) Distributions of the number of prophages in the bacterium separated by clonal groups and differentiating whether they have or do not have certain defense systems. A black box indicates that there is a significant difference, red if there are only genomes with absence of the defense system (or there is less than 1% of genomes per each clonal group with presence), and green if there are only genomes with presence (or there is less than 1% of genomes per each clonal group with absence), and the absence of a box indicates that there is no significant difference (Wilcoxon test, *P*-value <0.05). (**C**) Specific prophages occurring in bacterial genomes with specific combinations of defense systems. Each individual Circos plot is constructed from a single ST clonal group, indicated in the figure, using only those combinations found in at least 2% of the genomes of such clonal group (this threshold was used for clarity). The numbers around the circos indicate the number of genomes found with that prophage and that combination of defense systems. (**D**) The same as (**C**), comparing only the innate defense systems Ssp and R-M throughout the entire pangenome (including all ST groups), using only those combinations found in at least 1% of all genomes. Above: genomes with R-M or Ssp as the only defense system; below: genomes with R-M, R-M and CRISPR-Cas, or Ssp as the only defense system.

To evaluate the efficiency of the defense systems to prevent phage integration into the bacterium, the total number of prophages present in genomes with a given system was compared. Unexpectedly, the most abundant systems (Ssp and Gao-Qat, mostly present in group 2) appeared with a greater number of prophages (average higher than three prophages), while the main defense system of group 1, R-M, had an average of less than two prophages (Fig. 5A). This low number of prophages is even more pronounced when the abortive system CBASS appeared, also frequent in group 1. However, another system frequent in this group, RosmerTA, was associated with a greater number of prophages, like the genomes of group 2. The latter could be due to the fact that genes from this specific toxin-antitoxin system were found in several versions of prophages: P1017 (1 out of 1,138), P1075 (42 out of 42), P1240 (79 out of 79), and P2140 (36 out 37), average expected value of 3e − 139, genes found by HMM profiles.

However, all these calculations could be biased by the different representation of *A. baumannii* clonal groups. Therefore, it was decided to perform this evaluation by separating again the genomes by the most frequent clonal groups. Thus, if we consider that a clonal group is a group of genomes that are taxonomically very close, small changes in them, such as the presence of a certain defense system, could be associated with a lower number of prophages, if that system is efficient against certain phages.

For the CRISPR-Cas systems, it was observed that in the ST25 group, there were only genomes with this defense system, while in the ST10, ST2, ST3, and ST499 groups, this system does not appear (Fig. 5B). However, in the ST79 clonal group, there are genomes with and without CRISPR-Cas system, with a significant difference in the number of prophages present, in favor of the presence of the defense system. Specifically, CRISPR-Cas systems are present in genomes with an average of three prophages, while the absence of the defense system occurs in genomes with an average of two prophages. The presence of a higher number of prophages also occurs in the ST2 group when PD-T, RosmerTA, and Ssp systems are present.

In the ST1 clonal group, CRISPR-Cas system presence is related to a lower number of prophages, indicating the effectiveness and explaining the prevalence of this system in this clonal group. A fewer number of prophages is also associated with the presence of CBASS and RosmerTA in particular clonal groups.

Focusing on the innate systems, Ssp systems are associated with a higher number of prophages, as mentioned in the ST2 group. And, in clonal groups where R-M systems predominate, there is no significant difference between the number of prophages when R-M is present or absent, except in the case of ST79 and ST10 groups where again the presence of R-M is associated with a higher number of prophages in the bacterial genome.

Differences in the number of prophages depending on the presence or absence of a certain defense system could be due to the presence or absence of specific prophages (sensitive or resistant to that defense system). To test this, the presence of the most frequent prophages in each clonal group was evaluated versus the combination of defense systems in their genomes (Fig. 5C). Thus, in the ST1 group, the CRISPR-Cas system is associated with the presence of many of the most frequent phages in this clonal group. Therefore, the quantitative difference between presence and absence of the CRISPR-Cas system that can be seen in Fig. 5B would be due to the rest of prophages that are not frequent in this group.

Another case is the ST25 group, in which the presence of the PD-T4 system is associated with the presence of seven different prophages, and at the same time is clearly associated with the absence of prophages P1059, P1114, and P1183. In the ST2 group, each different combination of defense systems is related to the presence of different phages, except for the prophage DgiS1, which always appears, regardless of the defense systems of the bacterium. And in the ST3 group, the absence of the PD-Lambda system is related to a lower occurrence of the most frequent prophages.

On the other hand, in the ST79 group, the presence of CRISPR-Cas systems is associated with the presence of seven different prophages, whereas prophage P1165 does not appear together with the defense system (Fig. S4). This suggests that the CRISPR-Cas system may not prevent the presence of certain prophages, but it may prevent the presence of the prophage P1165, which is not avoided by the R-M systems.

Other interesting cases are the ST10 and ST78 groups. In the ST10 group, the presence of PD-T systems seems to prevent the occurrence of most of the frequent prophages in that group. This fact is reflected in the differences in the total number of prophages in genomes with the presence of the aforementioned systems in this clonal group, with a lower number of prophages being observed when the bacteria are armed with these systems (Fig. 5B). Finally, in the ST78 group, the presence of the PD-T and Gao_Qat systems seems to prevent the infection of most of the common prophages, as can be seen in the total number of prophages.

Furthermore, when comparing the collection of prophages present and absent in genomes that have only one of the two innate defense systems, R-M (with or without CRISPR-Cas) and Ssp, the prophage profile they exhibit is completely different (Fig. 5D).

### Prophages are positively and negatively associated with each defense system with different strength

We have already seen that certain prophages appeared to be related to specific defense systems (or combinations of them). Now, we aimed to assess the relevance of each prophage individually—that is, whether the presence or absence of specific prophages alone, without considering the others, can reliably indicate whether a bacterial genome harbors a given defense system. This approach would suggest that individual prophages may have different degrees of weight or importance in the maintenance (or gain-loss) of this defense system in the bacterial genome. This importance could be due to two possible relationships: negative (the prophage is rare when the defense system appears, and therefore suggests that the system avoids prophage infection) or positive (the prophage is very frequent when the defense system appears, and therefore suggests that the system does not avoid phage infection, or the phage could be the carrier of the defense system).

To do this, we created a predictive model, using machine learning techniques, to calculate the importance or weight of each individual prophage in tagging a bacterial genome with a given defense system. As a result of the evaluation of each model, different metrics were obtained that tested the performance (Fig. S5; Table S3). Thus, the best-performing models were those predicting the possession of the Ssp, CRISPR-Cas, Gabija, and R-M defense systems. And the models that performed worst were those that attempted to predict whether strains had the PD-T and CBASS systems.

**Fig 6:**
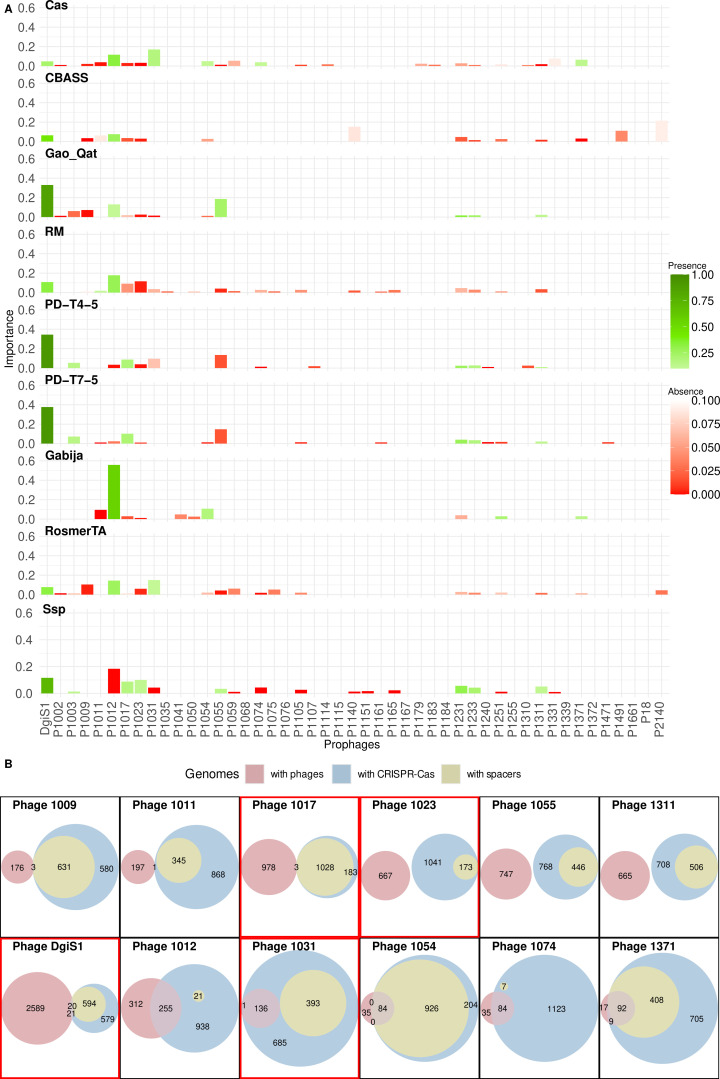
Machine learning approach to identify significant prophages associated with a specific defense system. (**A**) Importance of the different prophages in the classification of a genome as having a particular defense system. For each defense system, the importance of each prophage is shown by the height of the bars (prophages not important for a defense system do not display any bar). The relative abundance of prophages in genomes with a particular defense system is shown by colors: 0 to 0.1, red-white gradient, shows prophages absent or present at a very low frequency; 0.1 to 1, white-green gradient, shows high frequency prophages. (**B**) Number of genomes that contain a specific prophage, with separate categories highlighting those that have only the prophage, only CRISPR-Cas, or CRISPR-Cas systems with spacers against the prophage. It also includes combinations of these categories. The top row shows all prophages that are negatively correlated with the CRISPR-Cas system (importance >0.015 and relative frequency <0.1), and the bottom row shows those that are positively correlated (importance >0.04 and relative frequency >0.1). The Venn diagrams, highlighted by a red box, represent the prophages identified as important by machine learning analysis.

The results of this experiment showed that few prophages can be related to a given defense system, i.e., most prophages could be avoided by any defense system under certain circumstances (Fig. 6A). In the case of CRISPR-Cas systems, the low frequency of three prophages (P1011, P1017, and P1023) combined with the presence of two others (P1012 and P1031) allows us to predict with high confidence that this defense system is present. The presence of prophage P1012 is also important in predicting that the bacterium is armed, in addition to CRISPR-Cas, with the R-M, Gabija, and RosmerTA systems, which are three defense systems that co-occur with high frequency with CRISPR-Cas, as shown above. In addition, the lack of prophage P1023 is also important in predicting that a genome has R-M systems, as well as PD-T systems, RomerTA, and CBASS. In contrast, the presence of the prophage is important for predicting that the bacterial genome has the other innate system, Ssp.

In relation to P1055, it can be seen that it is an important prophage to predict the PD-T and Gao_Qat systems, but in an antagonistic way. While the PD-T systems probably avoid P1055, the Gao_Qat system is not able to avoid it, as both co-occur.

In fact, the two main innate systems present an almost inverse prophage profile, as previously seen. Specifically, the presence of prophage DgiS1, as well as prophages P1017 and P1023, together with the absence of prophage P1012, is associated with the presence of the Ssp system. However, the observed trend in prophages P1012 and P1023 suggests the presence of an R-M system within the bacterial genome. In fact, most of the prophages that are significant for predicting the presence of R-M systems are absent in genomes with this system, reinforcing the idea of the efficacy of this type of anti-phage defense. This is also the case with the CBASS system, highlighting the efficiency of this abortive system, as discussed in the previous section.

The prophage DgiS1 is a special case. It is a very frequent prophage in *A. baumannii*, which is not only important for predicting Ssp in the genome, but it also appears to be specially associated with the PD-T and Gao_Qat systems. PD-T systems are found inside the sequence of the prophage itself, while the second had already been found with a high frequency of co-occurrence previously, since this system shares integration site with DgiS1 (38). In the case of Ssp, the DgiS1 prophage does not carry the system itself, but it does bear the *sspD* gene of the defense system, which encodes an enzyme whose function is to add phosphorothioate groups. This could reflect a refined strategy to mark the phage genome and make it undetectable by the bacterial Ssp system. This gene was found in 63.6% of DgiS1 prophage genomes (expect value = 1.3e − 14 with HMM profile).

Other similar cases are the prophages P1075 and P2140, which showed importance in the prediction of the RosmerTA system. The complete RosmerTA system (genes encoding toxin and antitoxin proteins) has been found in the sequence of major versions of these prophages: 41 out of 42 in P1075, and 36 out of 37 in P2140. Therefore, the level of importance given by these prophages would be due to the fact that they provide the RosmerTA system to the bacterium.

The meaning of the results obtained by machine learning can be further probed for a specific defense system, the CRISPR-Cas. In this case, we can draw some predictions. If the absent phages are missing because they are fought away by the defense system, their CRISPR arrays must contain spacers that target the sequence of these prophages. Likewise, if the prophages are associated with the presence of the defense system, we should not find spacers, or if found, they would not prevent the presence of the prophage.

To verify this fact, we examined the number of genomes in which specific prophages appeared, together with the CRISPR-Cas systems and spacers that target the prophage in those systems, as well as the overlap of all of them. The result obtained was that for prophages negatively associated with CRISPR-Cas systems, the genomes in which the prophage appears have no CRISPR-Cas systems, and when this system appears, the prophage is not present, but the CRISPR arrays usually present spacers against the prophage (Fig. 6B). However, for prophages positively associated with CRISPR-Cas systems, the prophage appears to coexist with this defense system, and either the CRISPR arrays do not contain spacers targeting the phage, or if they do (as in the case of prophages P1031, P1054, and P1371), the prophage and spacers usually do not coincide in the same strain. This is so, except for prophages P1054 and P1371, where in many cases the prophage and the spacers against it coappear in the same bacterial genome. Of these last two prophages, the prophage P1371, precisely, presented a relatively high importance for classifying a genome as having CRISPR-Cas systems.

**Fig 7:**
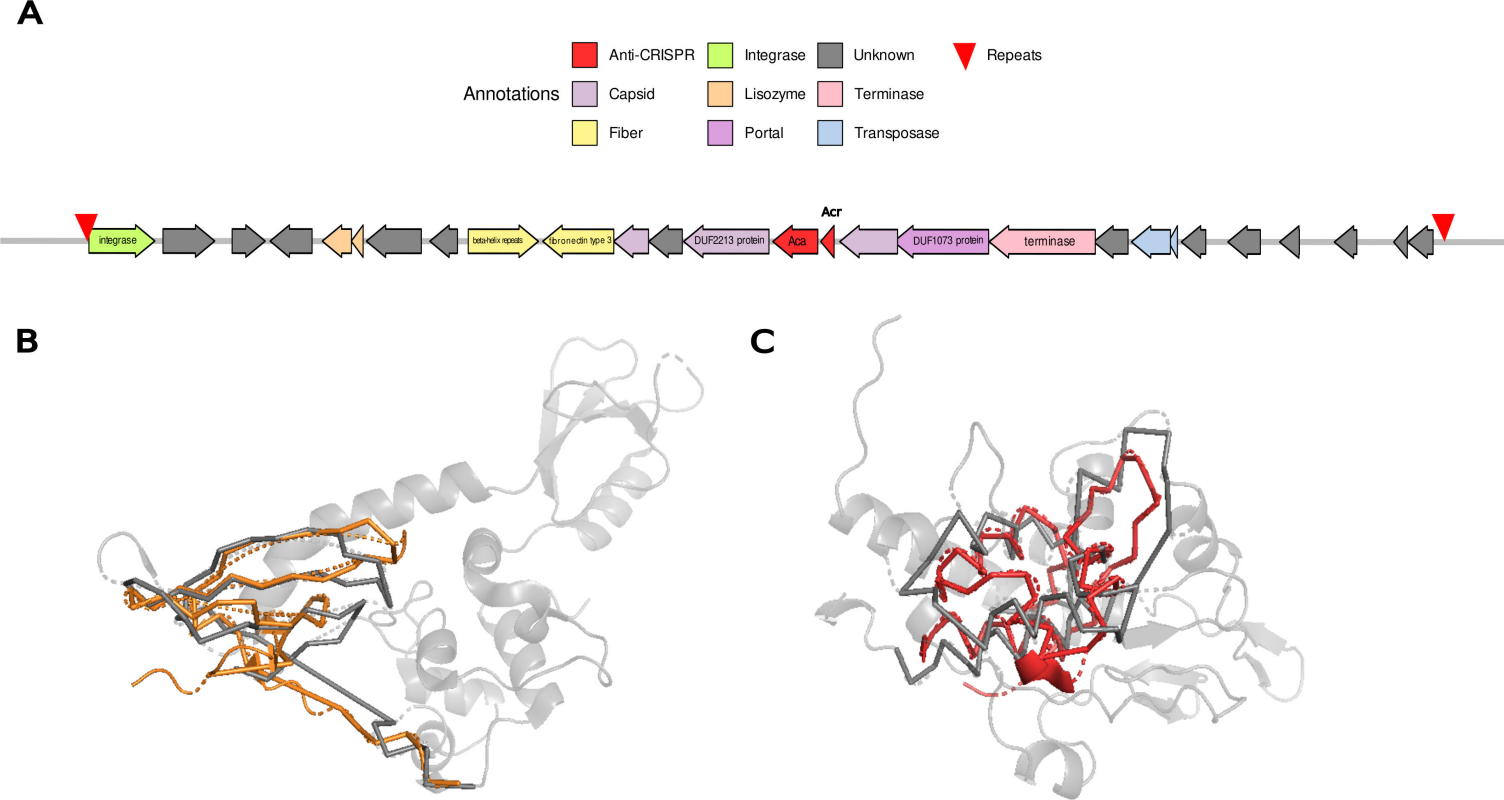
Genes and anti-CRISPR proteins of prophage 1012. (**A**) Coding sequences in the full sequence of P1012 together with their functional annotations. The putative phage integration site (direct repeats) is shown with inverted red triangles. The presence of the putative *acr* and *aca* in the sequence of P1012 is highlighted in red. (**B**) 3D alignment between the structural model of the protein encoded by the *acr* gene of P1012 (orange) and the structure of AcrIF 24 (PDB entry: 7XI1). The structural alignment has a TM score (Template Modeling score) of 0.4704, using the smaller protein structure. (**C**) 3D alignment between the structural model of the protein encoded by the *aca* gene of P1012 (red color) and the Aca1 structure (PDB entry: 7C0G). The alignment has a TM score of 0.4940 using the smaller protein structure.

The case of P1012 is an exception since this prophage appears in genomes that have CRISPR-Cas systems. However, the system shows almost no spacers against the prophage (only 21 genomes in the entire pangenome have spacers targeting this phage). Since the prophage itself does not carry a CRISPR-Cas system, possible anti-CRISPR systems were searched for in its sequence. To do this, a specific prediction of *acr* (anti-CRISPR genes) and *aca* (anti-CRISPR associated genes, usually found together with *acr* genes) sequences was made (39), validating the results by 3D structure similarity against proteins from characterized anti-CRISPR systems. Thus, a putative type I-F *acr* gene and its corresponding *aca* gene encoding a protein similar to Aca1 were found (Fig. 7). This anti-CRISPR protein could be preventing the generation of spacers, and therefore, the CRISPR-Cas activity.

## DISCUSSION

We have found 81 anti-phage defense systems in a pangenome of the species *A. baumannii*, none of them being found in all the genomes analyzed. However, the results suggest that these systems are available in the species, as a pan-immune system, being maintained in specific strains of environments where they suppose a competitive advantage (19). This idea is supported by the fact that a defense system in different strains that are close in evolutionary terms has different sequence variants, and closely related strains show divergent sequences, all of these suggesting horizontal gene transfer (40). We have also found a different prophage profile among different clonal groups of the bacterium, associated with a certain profile of defense systems.

Genomes analyzed showed an average of 3 ± 2 different defense systems, different from previous studies that, using the same methodology, found an average of 5 (41, 42). This difference could be due to the bias caused by the abundantly sequenced global clone 2, which, depending on the pangenome used, may have more or less weight in the final result, increasing the number of phages. In any case, the high number of unequally distributed defense systems throughout the *A. baumannii* pangenome would be correlated with the flexibility of this species (43), which could thus adapt to different environments dominated by different mobile genetic elements. Thus, some of these antiviral systems have a high frequency of coappearance, as we have found in the case of the *A. baumannii* CRISPR-Cas I-F and R-M type II systems. This fact has been described before and could be explained by a synergy that would improve the success of the former in creating new immunity to previously unrecognized phages (10, 11, 44). Spacer adaptation in CRISPR-Cas systems can be enhanced in cells also carrying R-M systems, as these would limit phage replication through degradation of their genome, and these excision products can be captured by the adaptation machinery of these systems (10). Likewise, the coexistence of CRISPR-Cas and R-M systems leads to a decrease in the frequency of spontaneous phage mutants that escape these defense systems (45). All this makes CRISPR-Cas systems one of the most efficient defense systems in preventing the gain of mobile genetic elements in bacteria, including prophages (40), and this is particularly remarkable in the genus *Acinetobacter* (17).

The appearance of specific systems in a genome may be attributed to the environment in which the bacterium is found, with the presence of certain phages acting as a selection pressure. Similarly, the coappearance of systems may be due to the same environmental constraints. Ssp was identified here as the most prevalent defense system. This system appears positively associated with the prophage DgiS1 of *A. baumannii* and PD-T systems, precisely because the genes that conform the PD-T systems appear within the sequence of the prophage, which may be responsible for its dissemination in the species (38). Ssp and Gao-Qat systems (the second most prevalent in the pangenome analyzed) are additional systems that appear to be positively connected to DgiS1. The Gao-Qat system is a defensive system that is found in the same defense archipelago where DgiS1 is integrated, next to a bacterial tmRNA gene. This may suggest synergy or symbiosis between both systems.

The innate Ssp and R-M systems in *A. baumannii* appear to have an antagonistic relationship. It is known that innate defense systems can show antagonistic relationships that prevent their stable coexistence, but the incompatibility can be overcome by epigenetic silencing, in which, precisely, these systems may be involved (46). Moreover, in this case, there would be two systems that label the bacterial DNA, so that only one of them would be sufficient for the proper functioning of an innate system. Here, we have shown that the presence of these two defense systems is strongly associated with certain clonal groups of the bacterium, which also exhibit specific prophage profiles. In fact, the clonal group associated with the Ssp system has a higher number of prophages than the groups with the R-M system, suggesting that this global clone 2, which is the most prevalent in hospital environments, could benefit from a more relaxed phage tolerance. This would lead to further infection and integration of new prophages, as these viral agents could contribute virulence factors or other defense systems to enhance the fitness and pathogenicity of the bacterium. It is interesting to note that the defense mechanisms possessed by these groups may actually contribute to their genetic divergence. For example, R-M systems have been shown to alter the rate of evolution in bacteria and to allow extensive genomic rearrangements and contribute to the genetic isolation of clonal groups (47, 48). In addition, genomes with the Ssp systems had a higher number of genes involved in antibiotic resistance. This reinforces the idea that these strains appear to allow horizontal gene transfer to their genomes to a greater extent than those with R-M systems. In fact, since this clonal group is enriched in clinical isolates, it would be expected to be more subject to selection pressure from antibiotic use.

Since the recent explosion in the number of known anti-phage systems, it has been shown that Ssp are rare defense systems that appear in only 2% of sequenced bacterial genomes (41, 42). However, a high occurrence of Ssp systems has recently been demonstrated in the *Acinetobacter* genus, appearing in one of the defense islands of their genomes (28). Using the protein SspB as a model, they observed independent sequence divergence that would suggest horizontal transfer. They also suggested that this prevalence would provide robust defense against phages in *A. baumannii*. However, they did not assess whether the system was limited to the global clone 2 strains in the species.

It is well known that ST2 strains, and other evolutionarily related strains, are included in a group called the international clone IC2 or global clone 2, the most isolated in outbreaks of *A. baumannii* (37). Despite their short divergence time, strains in this group present differences in their genomes, including different antibiotic resistance profiles (49), leading to distinct sublineages adapting to different environments or acquiring specific genetic traits in different regions (50). An important point to note about the results shown here for the Ssp system of *A. baumannii* is its extremely low similarity to this system in other species, including those of the same genus *Acinetobacter*. This suggests that this defense system has been recently acquired by the species, specifically by the ancestor of the global clone 2. In fact, the genes that form part of the Ssp system in this group appear in virtually all its strains, whereas they are almost non-existent in genomes outside the group (appearing in only 10 genomes). This once again supports their recent appearance in *A. baumannii* and suggests their importance in the establishment of this clinically important group. Another point of interest is the absence of the cognate nuclease in this system. It could be located in the same genomic region containing several global clone 2 signature genes, including a two-component system with a histidine kinase. This could suggest a new variant of the Ssp system or a role in epigenetics, similar to those already described in Dnd innate systems, also based on phosphorothioate modification (51).

Apart from *Acinetobacter*, the Ssp system is not prevalent at all. In two *Pseudomonas aeruginosa* pangenomes, no Ssp systems have been found in more than 3% of the strains (52, 53). In a recent pangenome of more than 1,300 *E. coli* strains, only three Ssp systems were found in its 41 defense hotspots, located in integrative and conjugative elements and integrative and mobilizable elements (26). Furthermore, in another Enterobacteriaceae, *Salmonella enterica* subsp. *enterica* serovar Typhimurium, while 44% of the more than 10,000 genomes analyzed had R-M systems, Ssp systems were not found (54).

Even in metagenome-assembled genomes, the frequency of Ssp systems is always around 2%, while R-M systems appear in 75% of soil communities, or 100% of those isolated in the human intestine (55). In addition, these defense systems do not appear in pangenomes of gram-positive bacteria such as *Staphylococcus haemolyticus* (56).

The R-M systems, considering all the types that encompass them, seem to be the innate system with the highest efficiency in avoiding phage infection, due to its prevalence in prokaryotes (4). However, the success of R-M systems may also be linked to their low maintenance cost, since they typically consist of only two genes (5). In addition, these defense systems are usually mobile and have a high degree of competition between them, which promotes their variability (57). Here, we have found 550 different pangenome genes associated with R-M systems. This high number of genes suggests that the system is subject to constant evolution, resulting in the emergence of variants that overcome the phage defenses (58). The appearance of R-M systems, even without the coappearance of any other, causes the bacteria to have none or very low numbers of prophages.

Phages can carry defense systems. This has been demonstrated in *P. aeruginosa* with defense systems such as R-M type IV and Wadjet (40), and abi and CBASS in other studies (17). In other cases, the defense system may coexist with the phage because the phage has acquired immunity to the defense system (59). All this has been contrasted in the analysis performed by machine learning, with the especially remarkable fact that several prophages seem to be avoided by R-M and CRISPR-Cas systems, while others seem to positively correlate with their appearance. In contrast, the same prophages have the opposite tendency in genomes presenting Ssp. These results could be supported in the case of CRISPR-Cas systems, thanks to the analysis of the spacers. All this demonstrates the usefulness of this type of analysis in microbiology (60).

We have shown, by means of machine learning modeling, that by taking into account the prophage profile, it is possible to predict which defense system a given strain of bacteria will have. This information is relevant for the use of phages to treat bacterial infections or phage therapy (61). When analyzed for lytic phages, the antiviral systems in the oyster-colonizing bacterium *Vibrio crassostreae* have been shown to be important in determining the phage-host range (62). However, in *E. coli*, genes encoding membrane proteins that are used by phages as adsorption factors or for the entry into the bacterial cell have been found to be more crucial in determining the bacterial strain-phage interaction, while the defensome would be less relevant in predicting this interaction (63). Using a different approach, we have shown here that antiviral systems could indeed modulate the bacterial phageome, considering the phages already integrated into the bacterial genome. All this, added to the defense systems that phages occasionally carry, could be shaping and constraining the mobilome of the species.

## MATERIALS AND METHODS

### Pangenome construction

The pangenome used was derived from 9,696 genomes belonging to the *A. baumannii* species in Rubio et al. (36). Each of these genomes has been structurally annotated, along with the reference genes inherent to the pangenome.

To eliminate redundancy, the skani tool v.0.2.2 was used to calculate the average nucleotide identity (ANI) by comparing all strains with each other (64). Subsequently, the groups of strains that shared 100% identity and coverage at the nucleotide sequence level were detected and eliminated, leaving only one strain as the reference of the group.

Of these remaining genomes, we discarded those that have a poor quality as *A. baumannii* genomes. To do this, we used the number of genes shared with the rest of the *A. baumannii* strains, in addition to the phylogenetic distribution. All genomes that had a number of average shared genes lower than 2.650 in the pangenome and/or belonged to an outgroup branch of our phylogeny were removed from the pangenome. Thus, 8,929 genomes were kept.

These genomes have an average size of 3,767 Mb and are fragmented into an average of 109.45 ± 100.89 sequences, with 286 of them completely closed. As an indication of the quality of these genomes, the NG50 of the assembly was calculated using the QUAST algorithm (65) and the strain ATCC 19606 as reference sequence (NCBI Genome: GCA_009035845.1). This NG50 value is referred to as the sequence length of the shortest fragment at 50% of the total genome length. In this way, an NG50 of 288,077 ± 743,592 was obtained, which, together with the low fragmentation, ensures only a reduced loss of genes.

To address redundancy, the average percentage of identity and coverage was calculated for these 8,929 genomes. An average percentage of identity of 98.05 ± 0.98 and coverage of 87.26 ± 5.76 were obtained. Considering the average genome size, there is an average difference of 75.34 kb in terms of sequence identity and a difference of 452.04 kb in terms of genome length.

The genes involved in antibiotic resistance were obtained from Pathogen Detection, which uses AMRFinderPlus (66).

### Defense system prediction

Defense systems were predicted in all genomes of *A. baumannii* using Defense Finder version 1.0.9 (41). Completed systems were considered, but isolated proteins containing HMM domains associated with defense systems (e-value ≤1e − 5) were also included in the analysis. Once the antiviral systems of *A. baumannii* were obtained, the defensome was constructed by mapping the genes that compose each system with reference genes in the pangenome. Genes found in more than 99% of the strains were considered core genes present in virtually all genomes of the species and therefore would not produce differences across genomes.

For further analysis, we used only those defense systems present in at least 1% of total strains in some cases, and the most frequent systems in others, to characterize the generalities from the bacterial species.

### Prophage prediction

Prophages were predicted in all genomes of *A. baumannii* using Phigaro version 2.3.0 with default parameters and the abs mode (67). Only prophages with a sequence longer than 9 kb were considered. All obtained prophages were clustered by similarity using MeShClust version 3.0 program with the identity threshold of 0.90, the value of total initial sequences as the parameter –v, and 1/4 of total initial sequences as the parameter *–*b.

To combine similar prophage genomes with the sequence oriented in the two possible reading directions, we performed a similarity search with BLASTN 2.10.0+ (68) by comparing the reference sequences of each cluster with each other. Then, clusters whose reference sequence shared at least 90% identity and coverage in both sequences were combined. In addition, if a reference sequence from a cluster shared at least 95% identity and 100% coverage with a larger reference sequence from another cluster, these clusters were also combined to join possible fragments from the same prophage.

Using this protocol, we were able to identify a total of 2,144 prophages. To analyze all prophages and discard potential false positives, we considered prophages present in 0.1% of the strains (present in at least nine genomes), leaving a total of 357 prophages. For further analysis, we used the prophages that were present in at least 10% of the strains of each major phylogenetic group of interest. For this purpose, multilocus sequence typing (MLST) was used with the Pasteur scheme. Thus, a total of 50 prophages were obtained, along with the addition of phage DgiS1, which we had previously shown to compete with a bacterial CRISPR-Cas I-F system for the same integration site (38).

To avoid redundancy, the prophage sequences were also subjected to an ANI analysis using skani, followed by a similarity check by BLAST (Fig. S6). Thus, we combined the redundant prophages, resulting in a total of 351 different prophages (present in 0.1% of the strains), of which 47 were selected as the most frequent (present in at least 10% of the genomes of each major phylogenetic group).

### Molecular phylogeny

To construct the molecular phylogeny, the amino acid sequences of genes present in 99% of the analyzed genomes were selected. This resulted in a total of 589 core proteins. The sequences were then joined by strain and aligned with the MAFFT v.7.271 program using the E-INS-i option (69), which is suitable for alignments with a large number of strains and gaps. Subsequently, the alignment was refined by removing areas with numerous gaps, few informative sites, and duplicate sequences using the ClipKIT v.2.2.4 algorithm with the KPI (Keep Parsimony-Informative sites) method (70). The molecular phylogeny was created using the IQ-TREE v.2.3.4 tool with the VT+R10 model and a bootstrap of 1,000 (71). The optimal model was calculated using the integrated ModelFinder algorithm in IQ-TREE. The phylogeny was represented using the R package ggtree v.1.10.5 (72).

The average branch length obtained was 0.0034 ± 0.0134, in a total of 13,227 branches (maximum length 0.9356, minimum 0.000001). This means an average of 1.34 changes per position in each of the 589 proteins used.

### Correlation between phylogenetic distance and sequence divergence

To study whether horizontal gene transfer exists, variants and distances from core genes used to categorize global clones (MLST) were used, as well as defense system gene sequences. The variants were obtained from the sequence of the gene sets that constitute each of the main defense systems. The sequences of these genes were assembled and grouped into clusters with a threshold of 95% identity and 90% coverage using CD-HIT v.4.8.1 software (73).

Variants of the MLST genes were identified using the MLST ST1 genes as a reference. The genes were then concatenated in the same order to obtain a unique sequence for each genome. Genomes lacking at least one complete MLST gene were removed. Phylogenetic distances were obtained using the distances calculated during the construction of the molecular phylogeny with the IQ-TREE tool.

To obtain the distances between the sequences of both core genes and defense system genes, all these sequences were aligned with the MAFFT v.7.271 program using the L-INS-i option (69). The alignment was processed using the ClipKIT v.2.2.4 algorithm with the KPI method (70) in order to obtain only informative regions. From the processed alignments, distances were obtained using the distmat tool with the nucleotide method of Kimura from the EMBOSS package (74).

Both phylogenetic and inter-sequence distances were plotted for each of the genome pairs, using a sampling of 10% of the points to be plotted. Density points were calculated using the 2D kernel density estimation method of the geom pointdensity v.0.2.0 package from R language. The statistical model used to calculate the scatter plot trend was the generalized additive model from the geom_smooth package.

### Defense system-prophage associations

The aim of this study was to search for phages that explain the presence or absence of a particular defense system. Therefore, we sought to employ machine learning classification models of defense systems using prophages as input features to ascertain the importance of these prophages in the classification and to be able to establish deep, meaningful associations between systems and prophages.

### Machine learning data preparation

The most frequent defense systems were used as target labels. The 47 most prevalent prophages in the pangenome were used as binary input features of the models, being a presence-absence matrix. Each row represents a genome, with columns indicating prophage presence (1) or absence (0), and associated metadata specifying the presence of particular defense systems.

The data set was randomly divided into training and test subsets, with a ratio of 70:30. The training set was used exclusively to build the model and optimize the hyperparameters, while the remaining 30% was reserved for the final evaluation of the model in order to avoid information leakage.

Machine learning models were implemented in R version 4.3.2 using the xgboost package (v.1.7.7.1). For each frequently occurring defense system, a separate XGBoost binary classification model was constructed. Feature importance was extracted to quantify how informative each prophage feature was for the prediction task. To optimize model performance, a grid search was performed over 10,000 randomly selected combinations of hyperparameters, including: eta (learning rate): {1e − 3, 1e − 2, 1e − 1}; max_depth: {1, 5, 10}; min_child_weight: {1, 5}; subsample: from 0.5 to 1.0 (step = 0.15); colsample_bytree: from 0.5 to 1.0 (step = 0.15); Regularization: gamma: {1e − 3, 1e − 2, 1e − 1, 1, 5}, lambda: {0, 1e − 3, 1e − 2, 1e − 1, 1, 10}, alpha: {0, 1e − 3, 1e − 2, 1e − 1, 1, 10}.

The “binary:logistic” objective was used to model the presence or absence of a defense system. Hyperparameter tuning was performed via fivefold cross-validation within the training set. That is, the training set was further partitioned into fivefolds: in each iteration, fourfolds were used for model fitting and one for validation. The combination yielding the lowest cross-validated error was selected.

As part of the grid search and hyperparameter optimization, subsampling parameters (subsample and colsample_bytree) were tuned to control overfitting and enhance model robustness. The subsample parameter controls the fraction of the training data randomly sampled for each boosting iteration, while colsample_bytree specifies the fraction of features (prophages) used per tree. By using values between 0.5 and 1.0, the model benefits from injected randomness, improving the generalization capacity of the model and reducing variance without significantly increasing bias.

Finally, the best model for each defense system was trained using the entire training set, with the most appropriate hyperparameters for each model.

### Model evaluation and interpretation

Once the hyperparameters had been tuned, the performance of the optimized model was evaluated on the independent testing set (30%). This provides an unbiased estimate of the model’s predictive performance. The performance metrics considered were accuracy (%CC), proportion of correct predictions; kappa, agreement between predictions and true labels, adjusted for chance; area under the ROC curve (Receiver Operating Characteristic curve), discriminatory power of the model; sensitivity (or recall) and specificity, which evaluate performance of a classification model.

These model performance metrics are shown in Fig. S5 (ROC curves of the different models) and summarized in Table S3.

### Protospacer search

The set of spacers was extracted with CRISPRCasFinder 4.2.20 (75) from the available genomes. These were compared against prophage sequences for protospacer search, using the BLASTN 2.10.0+ algorithm with the blast-short option for short sequences and a threshold of 90% spacer identity and coverage.

### Prophage annotations

To obtain the complete sequence of the prophage P1012, we used the raw data (SRA:ERR4686449) from the host bacterial genome to reassemble a bigger contig with the complete prophage. We searched for direct and inverse repeats to define the limits of phage integration. To do this, we looked for sequence repeats that appeared between the tRNA (putative insertion site) and the integrase of the phage using BLASTN.

Prophage genes were annotated using the PHASTEST (76), eggNOGmapper v.2 (77) tools, and the PhageScope database (78). Genes that remained unannotated were attempted to be annotated in depth using the BLASTP tools with the non-redundant protein databases of NCBI and IMG/VR 4.1 (79).

### Anti-CRISPR discovery

Anti-CRISPR (Acr and Aca) proteins were predicted using the AcrHub web server with default parameters and thresholds, which encompasses predictive tools such as PaCRISPR or AcrRanker, as well as HMM models (80). The 3D structures of the predicted anti-CRISPR proteins were modeled using AlphaFold 3 (2025.01.28) (81), and these were aligned using USalign tool v.20240730 (82) versus the following PDB structures: 6KYF, 7DTR, 7ELM, 7ELN, 7WE6, 7XI1, and 7YHS (anti-CRISPR-IF, AcrIF); 7C0G and 7FA3 (Associated-CRISPR protein, Aca1). Only structure alignments representing complex secondary structures and whose TM score (Template Modeling score) was greater than 0.4 were taken into account. Structural alignments were visualized using PyMol v.3.1.3.

### Statistics

Data were analyzed using R version 4.3.2. Normal distribution of the different sets of data was assessed with the Shapiro-Wilk test. Statistical significance between different groups was determined using Wilcoxon and Kruskal-Wallis tests, where *P*-value lower than 0.05 was considered significant. Standard deviation was used to determine the error ranges when dealing with average data. Adjustments to *P*-values for multiple testing were made using the Bonferroni method.

## Data Availability

The analyzed pangenome was constructed in a previous work and is available in the following repositories: https://zenodo.org/record/7224593#.Y0-_KExBxPZ, https://github.com/UPOBioinfo/cancun/. The data generated in the present work are all available in the Zenodo code repository from the following section. All the code for the creation of the defensome, the data analysis and the realization of the figures in this article, including the input datasets, is written in Python 3.12.2 and R version 4.3.2 programming languages, and is available in the following Zenodo repository: https://doi.org/10.5281/zenodo.15871372.

## SUPPLEMENTAL MATERIAL

Supplemental Figures mbio.02135-25-s0001.pdf Figures S1 to S6.

Table S1 mbio.02135-25-s0002.xlsx Strains of analyzed *A. baumannii*.

Table S2 mbio.02135-25-s0003.xlsx Defense systems and their genes from the pangenome of *A. baumannii*.

Table S3 mbio.02135-25-s0004.xlsx Machine learning performance and hyperparameters.

ASM does not own the copyrights to Supplemental Material that may be linked to, or accessed through, an article. The authors have granted ASM a non-exclusive, world-wide license to publish the Supplemental Material files. Please contact the corresponding author directly for reuse.
