# Supplementary material for: The evolutionary replacement of restriction-modification by Ssp antiviral systems is associated with the distribution of prophages in the major clonal group of Acinetobacter baumannii
Source: mBio. 2025 Aug 18;16(9):e02135-25. doi: 10.1128/mbio.02135-25 (PMC12421847; doi:10.1128/mbio.02135-25)
Supplement: Supplemental Figures — Figures S1 to S6. [file mbio.02135-25-s0001.pdf]

The evolutionary replacement of restriction-modification by Ssp antiviral systems is associated with the distribution of prophages in the major clonal group of *Acinetobacter baumannii*

Supplementary Files

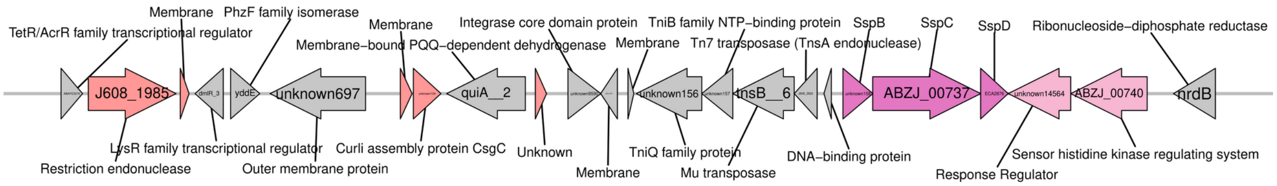

Fig. S1. Locus where the Ssp system is mostly found. Genes highlighted in colors other than gray appear in more than 90% of strains in group 2 and in less than 1% of strains in group 1.

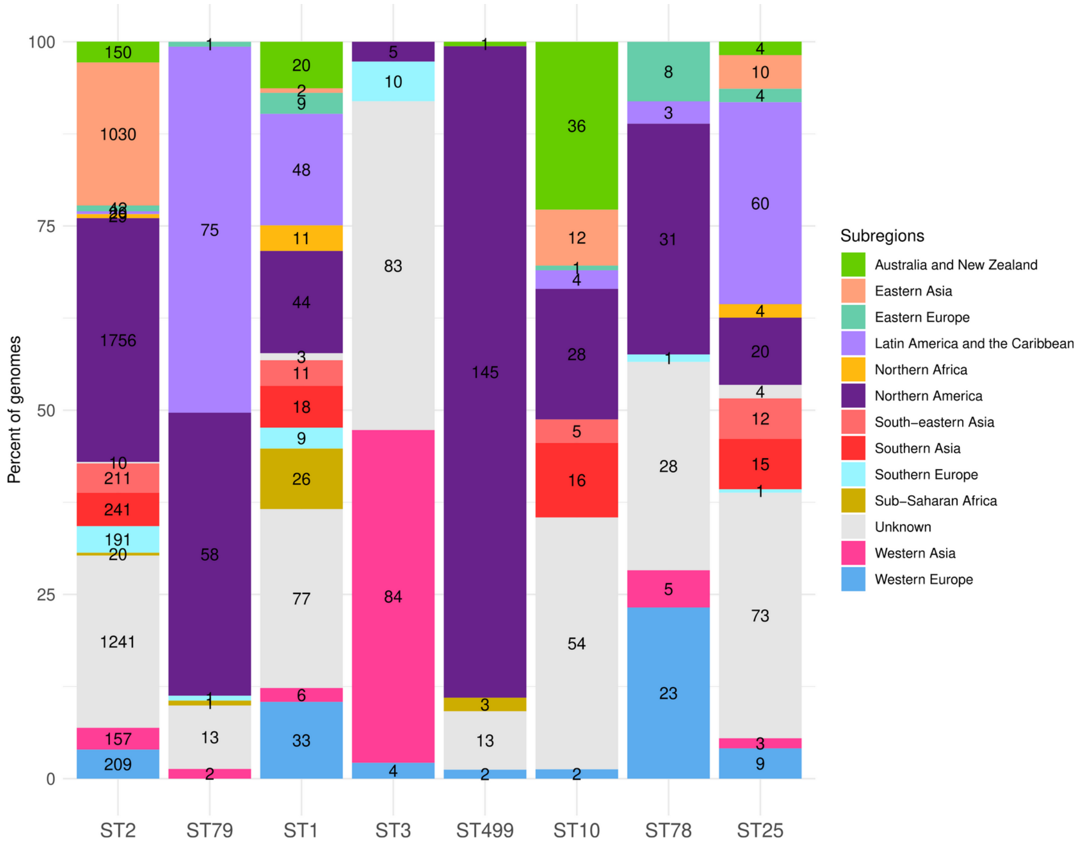

Fig. S2. Distribution by subcontinent of the different MLST groups.

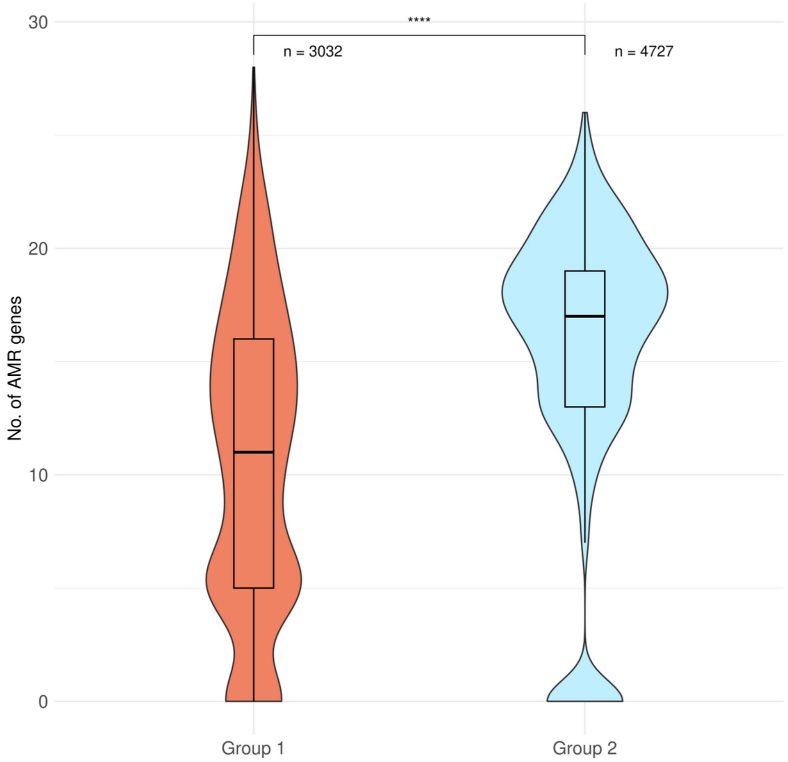

Fig. S3. Distribution of the number of genes involved in antibiotic resistance in groups 1 and 2. Wilcoxon, p-value = 2.32e-219.

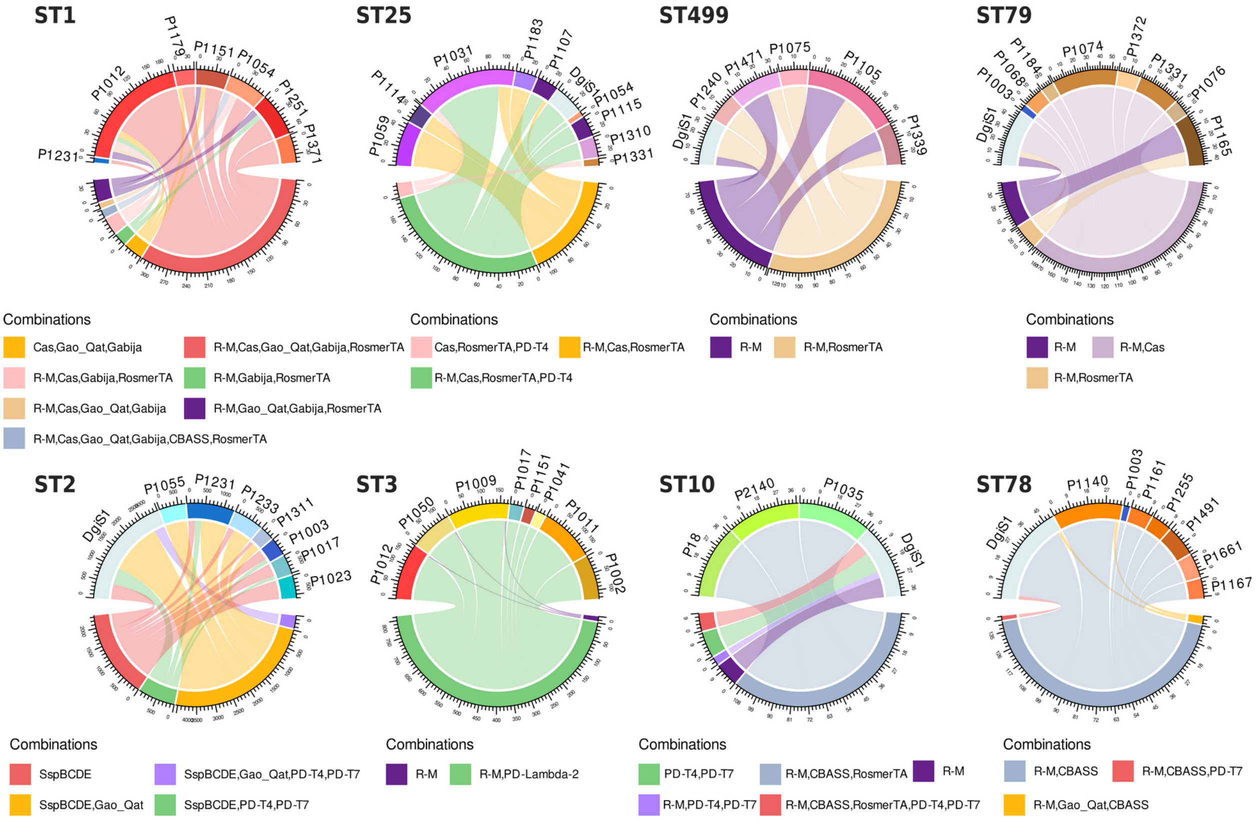

Fig. S4. Specific prophages occurring in bacterial genomes with specific combinations of defense systems (clonal groups missing in Fig. 4C).

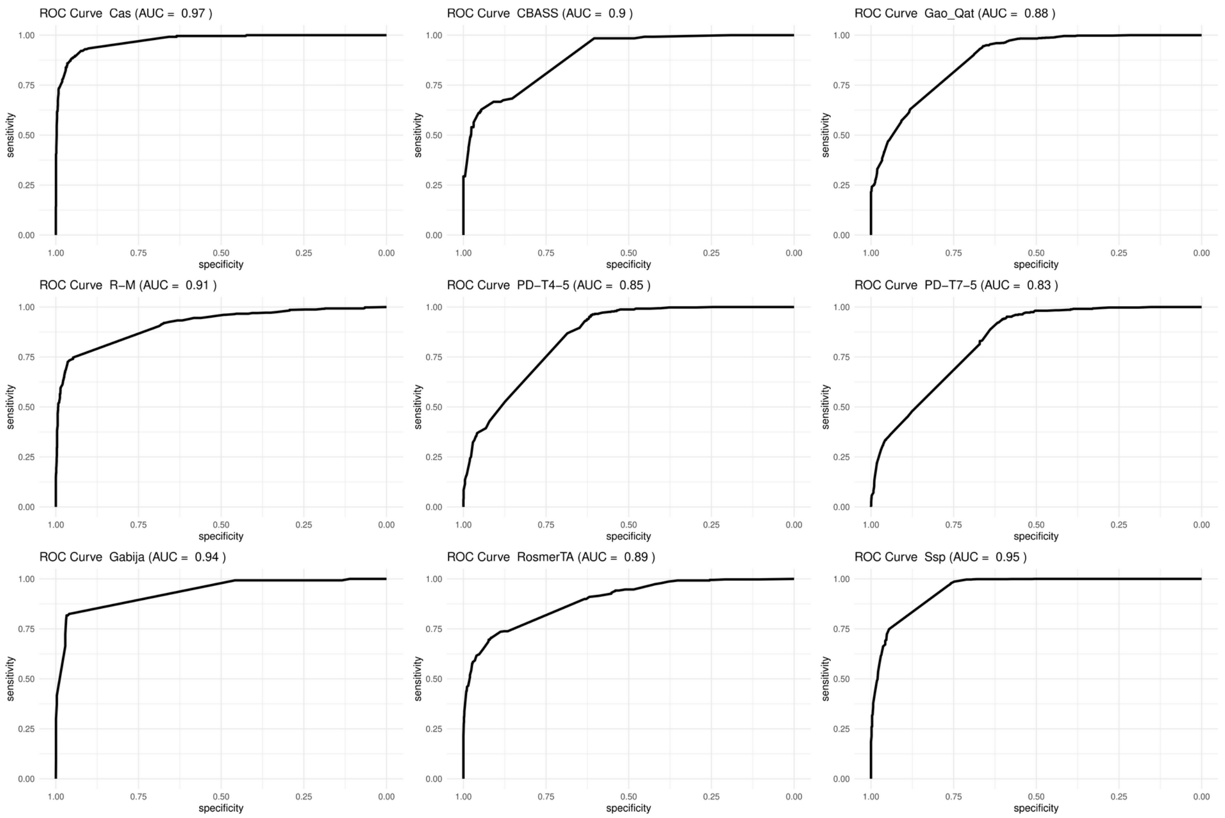

Fig. S5. ROC curves for each of the models used for each defense system. The models with the best performance are those associated with the CRISPR-Cas, Ssp, Gabija and R-M systems.

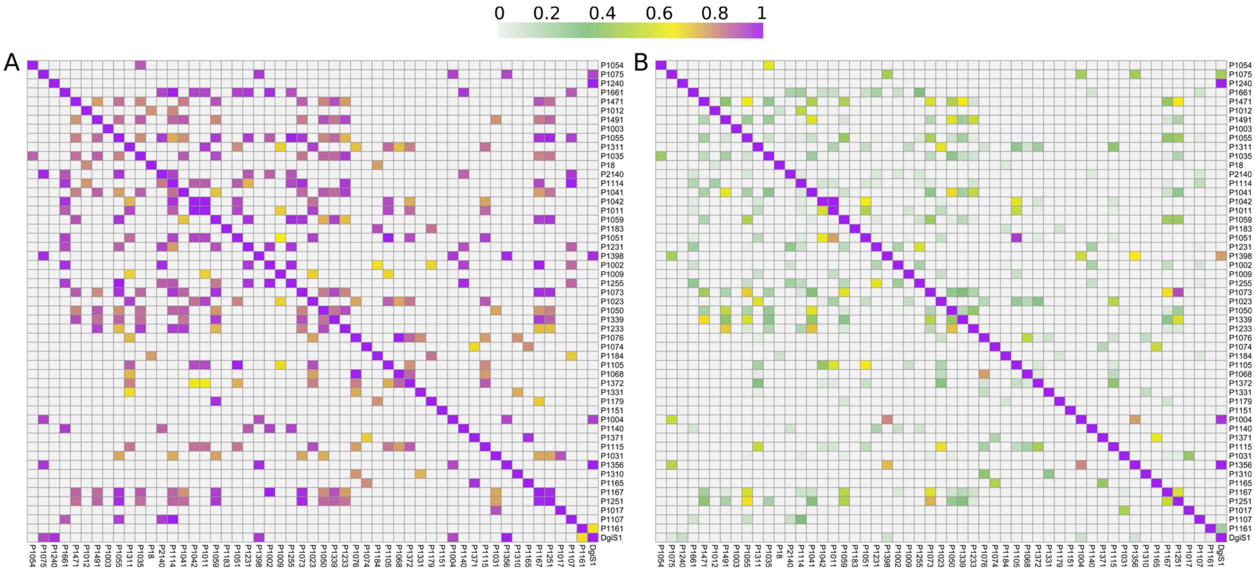

Fig. S6. Average nucleotide identity (ANI) of the prophages found in the bacterial genomes.
